# Dynamic Visualization and Quantification of Single Vesicle Opening and Content by Coupling Vesicle Impact Electrochemical Cytometry with Confocal Microscopy

**DOI:** 10.1021/acsmeasuresciau.1c00021

**Published:** 2021-08-09

**Authors:** Ying-Ning Zheng, Tho D. K. Nguyen, Johan Dunevall, Nhu T. N. Phan, Andrew G. Ewing

**Affiliations:** Department of Chemistry and Molecular Biology, University of Gothenburg, Kemivägen 10, 41296 Gothenburg, Sweden

**Keywords:** Vesicle impact electrochemical cytometry, confocal microscopy, vesicles, fluorescence, exocytosis

## Abstract

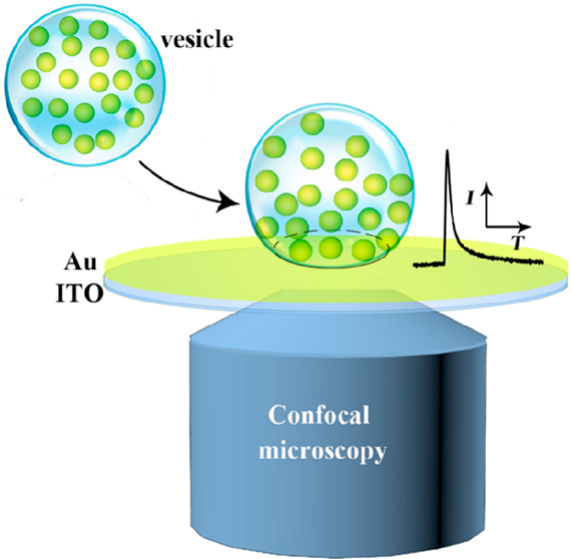

In this work, we introduce a novel method for visualization and quantitative measurement of the vesicle opening process by correlation of vesicle impact electrochemical cytometry (VIEC) with confocal microscopy. We have used a fluorophore conjugated to lipids to label the vesicle membrane and manipulate the membrane properties, which appears to make the membrane more susceptible to electroporation. The neurotransmitters inside the vesicles were visualized by use of a fluorescence false neurotransmitter 511 (FFN 511) through accumulation inside the vesicle via the neuronal vesicular monoamine transporter 2 (VMAT 2). Optical and electrochemical measurements of single vesicle electroporation were carried out using an in-house, disk-shaped, gold-modified ITO (Au/ITO) microelectrode device (5 nm thick, 33 μm diameter), which simultaneously acted as an electrode surface for VIEC and an optically transparent surface for confocal microscopy. As a result, the processes of adsorption, electroporation, and opening of single vesicles followed by neurotransmitter release on the Au/ITO surface have been simultaneously visualized and measured. Three opening patterns of single isolated vesicles were frequently observed. Comparing the vesicle opening patterns with their corresponding VIEC spikes, we propose that the behavior of the vesicular membrane on the electrode surface, including the adsorption time, residence time before vesicle opening, and the retention time after vesicle opening, are closely related to the vesicle content and size. Large vesicles with high content tend to adsorb to the electrode faster with higher frequency, followed by a shorter residence time before releasing their content, and their membrane remains on the electrode surface longer compared to the small vesicles with low content. With this approach, we start to unravel the vesicle opening process and to examine the fundamentals of exocytosis, supporting the proposed mechanism of partial or subquantal release in exocytosis.

## Introduction

Vesicles play an important role in synaptic signaling during neuronal transmission, as they are the major organelles for the storage and release of neurotransmitters.^1−3^ Quantification of intravesicular neurotransmitter content and understanding the dynamic release process is vital for studying the mechanism of neurotransmission and malfunction in neurodegenerative diseases. In recent years, electrochemical strategies based on micro/nanoelectrodes and multielectrode arrays have been developed for quantitative measurements of intravesicular content and real-time monitoring of their release dynamics.^4−7^ Our group has recently developed a technique, vesicle impact electrochemical cytometry (VIEC), allowing quantification of the catecholamine content inside single adrenal chromaffin vesicles as they adsorb and rupture on a 33-μm-diameter disk-shaped carbon electrode.^8^ These micro/nanoelectrochemical methods provide effective ways for accurate quantification of vesicular content with sub-millisecond temporal resolution that matches the fast vesicle release kinetic process. Hence, they offer an opportunity to understand the dynamics and mechanism of the vesicle opening process at the electrode surface, providing clues of how vesicles arrive, dock, and reside on the electrode surface, how the vesicular membrane changes during the opening, and how these characteristics relate to the content and the size of the vesicles.

Confocal laser scanning microscopy (CONF) is an optical fluorescence imaging technique with excellent spatial resolution. By using a pinhole to block out-of-focus light in image formation, the optical resolution is increased by CONF (approximately 200 nm).^9^ To obtain comprehensive and precise data of the opening mechanism of a single isolated vesicle on the electrode, a combination of VIEC and CONF can be used to obtain the electrical signals of the released content and simultaneously observe the behavior of the vesicular membrane during the vesicle opening.

The combination of VIEC and CONF is technically challenging because of the fundamental measurement differences between the two techniques. In VIEC, potential is applied at the electrode surface, leading to electroporation of the membrane and formation of an initial pore between the vesicle and electrode, out of which the neurotransmitter is released.^10−12^ Interestingly, due to different factors including protein distribution, lipid properties, and conformational change of vesicles on the electrode surface, not all adsorbed vesicles appear to form a pore, expelling their content; instead, in most of the cases, vesicles seem to stay intact.^13^ Thus, VIEC measurements are usually carried out at a high concentration of vesicle suspension, enabling more opening events to be determined. In contrast, for CONF live imaging, the vesicles placed on the observation region under the microscope should be well distributed and limited in number in order to be easily observed. In addition, VIEC measurements need to be carried out on a conductive electrode material, for example a disk-shaped carbon fiber electrode,^14−17^ whereas CONF imaging is performed on an optical-quality coverslip.^18,19^ In order to combine the two techniques, the electrode should be both transparent and electrochemically conductive. Indium tin oxide (ITO) thin-film-coated glass substrates have been widely used in optoelectronic devices that require good optical transparency over the visible region and high electrical conductivity, for instance, liquid crystal displays, flat panels, and organic light-emitting diodes.^20−23^ However, the electrochemical kinetics of molecules like catecholamines on ITO is slow. To improve the electrochemical property of the ITO electrode, the electrode surface can be modified with conducting and electrocatalytic metals, particularly gold, using different methods, such as self-assembly of gold nanoparticles,^24^ electrodeposition,^25,26^ vacuum sputtering,^27^ and thermal evaporation.^28^

In this paper, we present a novel method for visualization of the opening dynamics of single mammalian vesicles and quantitative measurement of their content using a combination of VIEC with CONF imaging on a gold-modified ITO microelectrode. Vesicles were isolated from the medulla of bovine adrenal glands. The vesicle membrane was labeled with 18:1 rhodamine-labeled phoshatidyl ethanolamine (PE) in order to increase the vesicle opening frequency, as was shown in previous work.^29^ To obtain high conductivity and electrocatalytic properties, a 5-nm-thick, 33-μm-diameter, disk-shaped, gold-modified ITO (Au/ITO) microelectrode was fabricated.^30,31^ The microelectrode simultaneously acted as a transparent substrate for CONF imaging and a microelectrode for catecholamine detection in VIEC measurements. Additionally, to visualize the vesicles, they were loaded with a fluorescent false neurotransmitter, a dye for targeting monoamine neurotransmitters, which are transported from the cytoplasm into synaptic vesicles via the neuronal vesicular monoamine transporter 2 (VMAT2).^32,33^ By correlating VIEC spikes with CONF images during the same time frame, we observed three main patterns of vesicle opening, which corresponded to the size and content of the vesicles.

## Experimental Section

### Reagents and Materials

Basic chemicals were obtained from Sigma-Aldrich and used as received. Fluorescence false neurotransmitter 511 (FFN 511) was purchased from Abcam Inc. 1,2-Dioleoyl-*sn*-glycero-3-phosphoethanolamine-*N*-(lissamine rhodamine B sulfonyl) (ammonium salt) (18:1 Liss Rhod PE) was purchased from Avanti Polar Lipids, USA. ITO glass (22 × 40 mm, #1.5) was purchased from SPI Supplies Inc. Homogenizing buffer (310 mOsm/kg) contained 0.3 M sucrose, 1 mM EDTA, 1 mM MgSO_4_, 10 mM HEPES, 10 mM KCl, and cOmplete Protease Inhibitor (Roche, Sweden). Lockes stock buffer (10×, pH 7.4) containing 1.54 M NaCl, 56 mM KCl, 36 mM NaHCO_3_, 56 mM glucose, and 50 mM HEPES was diluted by distilled water each time to obtain the 1× Lockes buffer for storage and rinsing the adrenal glands. Fresh bovine adrenal glands were kindly donated by Dalsjöfors Kött AB, Dalsjöfors, Sweden.

### Isolation of Chromaffin Vesicles

The isolation protocol for vesicles was based on the procedure developed by the Borges research group (University of La Laguna, Spain).^34^ The stock vesicle suspension was carefully diluted 10× in homogenization solution. The labeled vesicles were isolated from the freshly cut adrenal glands and resuspended in a homogenization buffer before being measured on the VIEC/CONF setup. For each measurement, 1 mL of vesicle solution suspension was added to a polylactic acid (PLA) chamber (1 × 2.5 × 0.5 cm) on a Au/ITO microelectrode device and let stand for 5 min for the vesicles adsorbing onto the electrode surface.

### Labeling Isolated Vesicles

FFN 511 solution (10 μL, 100 mM) was added to the 10 mL vesicle suspension, followed by incubation at 4 °C for 40 min to label the vesicle content. Then, the mixture was centrifuged at 10 000*g* for 20 min to remove the excess reagents. Subsequently, 142 μL of 18:1 Liss Rhod PE solution (1.5 mg/mL) was added to the vesicle solution (10 mL) and incubated at 4 °C for 15 min. The solution was finally centrifuged at 10 000*g* for 20 min to remove the excess reagents.

### Fabrication of Au/ITO Electrodes

A layer of 5 nm gold was deposited on an ITO-coated coverslip (SPI Supplies) by e-beam thin-film evaporation (Lesker). The surface was then cleaned with isopropyl alcohol and blow-dried with nitrogen gas. The coverslip was then spin-coated with a photoresist layer S1813 (SHIPLEY) at 4000 rpm for 30 s to yield a film thickness of about 1 μm and was then baked at 115 °C for 1 min. A 20-channel microelectrode array (MEA) pattern was developed, and this was transferred to the coverslip by UV lithography (KS MA6, Suss MicroTec) with a chrome mask of the 20-channel MEA design. After UV exposure, the electrodes were developed with MF319 developer (SHIPLEY) for 1 min with mild shaking followed by hardening by baking at 115 °C for 5 min on a hot plate and finally dry etching using an ICP argon plasma (Oxford Ionfab 300 Reactive Ion Beam). After dry etching, the photoresist was removed by placement in a low-intensity ultrasonication bath with mr-Rem 400 (Micro Resist Technology) at 50 °C for 20 min. To obtain a layer of insulation covering most of the structure except the probe area and the contact pad area, the glass wafers with MEAs were spin-coated with the photoresist SU-8 3005 (MicroChem) at 4000 rpm for 30 s to produce a film of the SU-8 3005 with a thickness of about 5 μm. The wafer was then baked at 65 °C for 1 min and at 95 °C for 3 min on a hot plate. The pattern for the insulated area was defined on top of the MEAs by UV lithography (KS MA6, Suss MicroTec) with a second chrome mask showing the probe area and the contact pad design and was subsequently baked at 65 °C for 1 min and 95 °C for 3 min on a hot plate. It was then developed with SU-8 developer mr-Dev 600 (Micro Resist Technology) for 2 + 2 min with a mild shaking. Finally, the device was baked at 150 °C for 10 min on a hot plate.

Afterward, a PLA chamber (1 × 2.5 × 0.5 cm) was prepared by 3D printing and attached to this SU-8 film on the glass wafer for the vesicle’s suspension reservoir. Electrical contact was achieved by manually placing connection pads onto the glass wafer.

### MEA Characterization

Electrochemical characterization of the MEAs was carried out with cyclic voltammetry by performing a voltage scan between the potentials of −0.5 and +0.2 V (vs Ag/AgCl reference electrode) in a solution of 5 mM Ru[NH_3_]_6_^3+^ in PBS buffer. The voltage was scanned between −0.2 and +0.8 V (vs a Ag/AgCl reference electrode) in a solution of 100 μM dopamine in PBS buffer (pH 7.4) using a 1030B multichannel potentiostat (CH Instruments). Steady-state voltammetric behavior was obtained for the Au/ITO MEAs.

The surface of the MEAs was characterized using scanning probe microscopy (SPM) (Bruker Dimension 3100). The thickness of the conductive material layer (Au, ITO) was examined with a line-scan using a surface profiler (Dektak). To characterize how well the light can pass through the MEA surface, we also tested the transmittance (%*T*) in the range of wavelength of 300–800 nm.

### Vesicle Impact Electrochemical Cytometry (VIEC)

Electrochemical detection of single isolated vesicle content was performed on the Au/ITO microelectrode as a working electrode at a constant potential of +700 mV (vs Ag/AgCl) operated by a potentiostat (Axopatch 200B, Molecular Devices). The recorded signal was filtered by 2 kHz with a four-pole Bessel filter and digitized at a 10 kHz sampling rate using a Digidata model 1440A with Axoscope 10.3 software (Axon Instruments Inc.). For each measurement, 1 mL of vesicle solution was placed on the Au/ITO microelectrode device in a polylactic acid (PLA) chamber (1 × 2.5 × 0.5 cm) and let stand still for 5 min so that the vesicles adsorb onto the electrode surface.

For the analysis, the initial spikes of VIEC data were identified, and the characteristic parameters of the spikes including the area, *t*_1/2_, and *i*_max_ as well as foot area and foot duration were determined using Igor Pro 6.37 software. Signals were designated as spikes if their *i*_max_ values were 5 × RMS noise for a 5-s portion of the stable baseline. All peaks identified by the program were inspected visually, and unusual peaks were manually excluded from the data sets. Further processing and data analysis related to confocal imaging such as defining time frames, grouping of spikes and frames were performed using in-house developed software written in MATLAB (Mathworks Inc.). A spike that occurred within a time frame (by time correlation) corresponding to the disappearance of a vesicle visualized by confocal imaging in the same time frame was assigned as a correlated event.

### Confocal Microscope Imaging

Confocal imaging was performed with an Abberior Expert Line STED microscope (Göttingen, Germany) in confocal mode. Rh-PE and FFN 511 were sequentially excited at 585 and 405 nm, respectively, and their fluorescence was collected at 575–650 and 460–550 nm, respectively. The confocal scanning was performed at the surface of the electrode zone with the XY mode (parallel to the electrode surface) and XYT mode with an imaging area of 30–40 μm^2^, a pixel size of 50–70 nm, and a time frame of 5 s. The confocal images were analyzed with ImageJ.

## Results and Discussion

### Characterization of Au/ITO Microelectrodes

**Figure 1:**
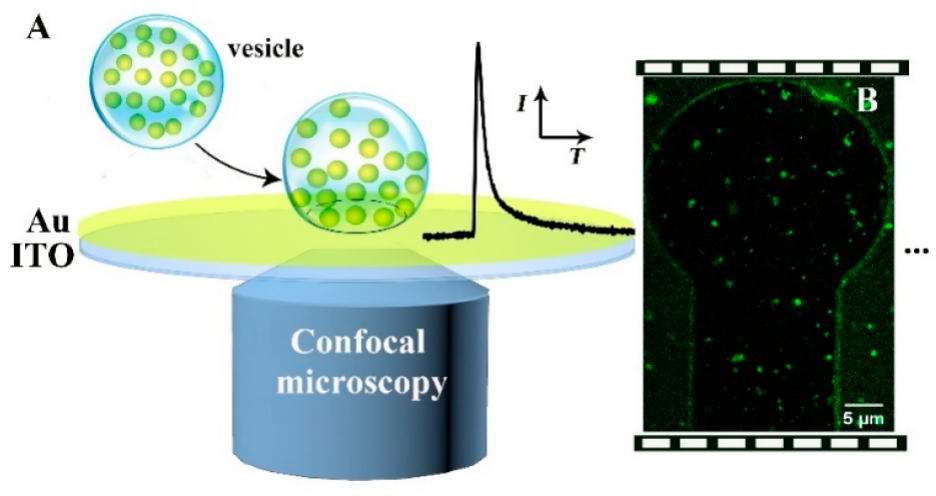
(A) Setup for CONF/VIEC correlation. (B) A CONF image of labeled vesicles on a Au/ITO electrode surface.

**Figure 2:**
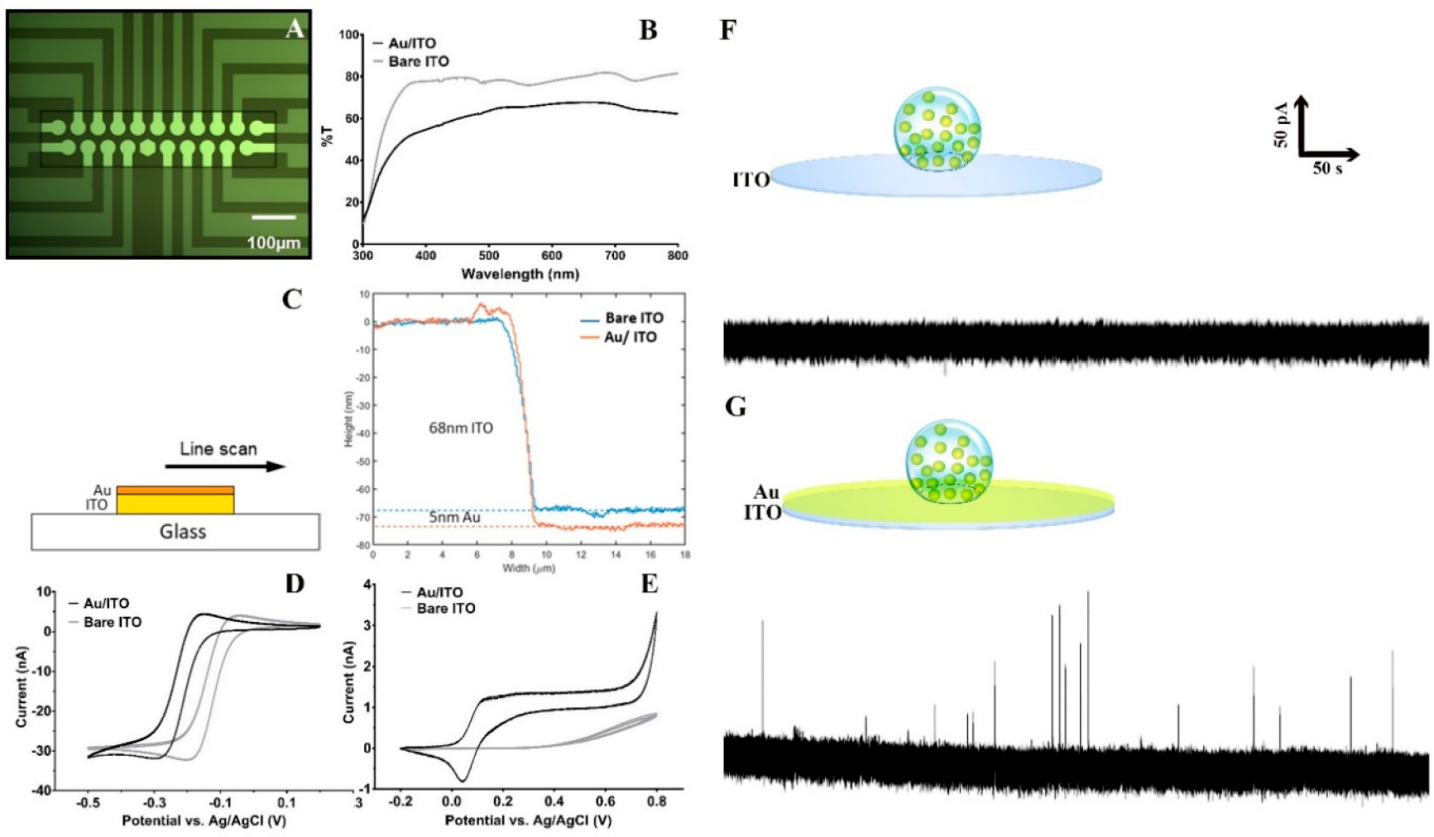
(A) Micrographs of one MEA area consisting of 20 microelectrodes (ø 33 μm). (B) Transmittance of bare ITO electrode (gray line) and 5 nm layer of Au modified on ITO electrode (black line) over the wavelength range of 300–800 nm. (C) Line-scan profile showing the thickness of a Au layer and ITO layer of a single microelectrode. Cyclic voltammograms (scan rate: 100 mV/s) were obtained in (D) 5 mM Ru[NH_3_]_6_^3+^ in PBS buffer (pH 7.4) and (E) 100 μM dopamine in PBS buffer (pH 7.4). The black curves show the voltammogram obtained with a Au/ITO microelectrode, and the gray curve shows the voltammogram obtained with a bare ITO microelectrode. Typical VIEC traces on an (F) ITO microelectrode and a (G) Au/ITO microelectrode. Electrode oxidation potential: +700 mV vs Ag/AgCl.

As shown in Figure 1, VIEC measurements and CONF imaging of isolated single vesicles are performed simultaneously. For each measurement, one of 20-channel MEAs was connected to the potentiostat and simultaneously visualized by CONF microscopy (Figure 2A). The implementation of the VIEC/CONF correlative measurement relies on the specific property of the working electrode, which simultaneously allows both fast electrochemical signal recording and fluorescence imaging. Thus, the materials used for the working electrode need to be both transparent and electrically conductive. We examined both ITO and Au/ITO electrodes as electrode substrates for these experiments.

The compatibility of the MEAs with both VIEC and microscopy heavily depends on the thickness and roughness of the Au layer on the ITO surface. A line-scan using a surface profiler (Figure 2B) showed that a Au layer of 5 nm was deposited on the ITO surface. Scanning probe microscopy (SPM) showed that a bare ITO-coated coverslip had a very flat surface (Figure S1A) with an RMS surface roughness *R*_q_ of 0.38 nm. Similarly, a 5 nm gold film deposited on top of the ITO glass using vacuum evaporation resulted in a flat surface (Figure S1B) with an RMS surface roughness *R*_q_ of 0.36 nm.

The electrode transmittance is a critical factor for the optical measurement. To characterize how well the light passed through the MEA surface, we measured the transmittance (%*T*) of light with wavelengths from 300 to 800 nm. The bare ITO surface had an average transmittance of 80%, whereas the 5 nm Au/ITO surface had an average transmittance of about 60%, and it is more transparent at red and far-red than blue wavelengths (Figure 2C).

Cyclic voltammetry was used to characterize the electrochemical performance of the Au/ITO MEAs. At potentials greater than +0.7 V vs Ag/AgCl reference electrode, we observed the onset of the oxidation of Au (Figure 2D); therefore, +0.7 V was used as the maximum potential in VIEC experiments. Compared to the bare ITO electrodes, the Au/ITO electrodes exhibited better electrochemical characteristics and higher response for both 5 mM Ru[NH_3_]_6_^3+^ (Figure 2D) and 100 μM dopamine (Figure 2E). The amperometric responses of the two electrodes were further tested with 18:1 Liss Rhod PE- and FFN 511-labeled isolated chromaffin vesicles in a homogenization solution. In Figure 2F, after a +700-mV potential was applied to the electrode, no spikes were detected at the ITO electrode in the vesicle suspension, while several spikes were detected from the same solution at the Au/ITO electrode (Figure 2G). Thus, it appears that the Au/ITO MEAs show good transparency and electrochemical properties for detection of dopamine in VIEC/CONF correlative measurements.

### Quantification of Vesicle Catecholamine Content by CONF/VIEC

In this work, we used rhodamine conjugated to phosphatidyl ethanolamine (Rh:PE), a fluorophore conjugated to vesicular membrane lipids, to fluorescently monitor the vesicles. It is thought that excited fluorophores in the lipid bilayer produce reactive oxygen species leading to the oxidation of vesicle membrane compartments.^36^ This also makes the membrane more susceptible to opening during the VIEC measurement, increasing the frequency of vesicle opening on the electrode.^31^

**Figure 3:**
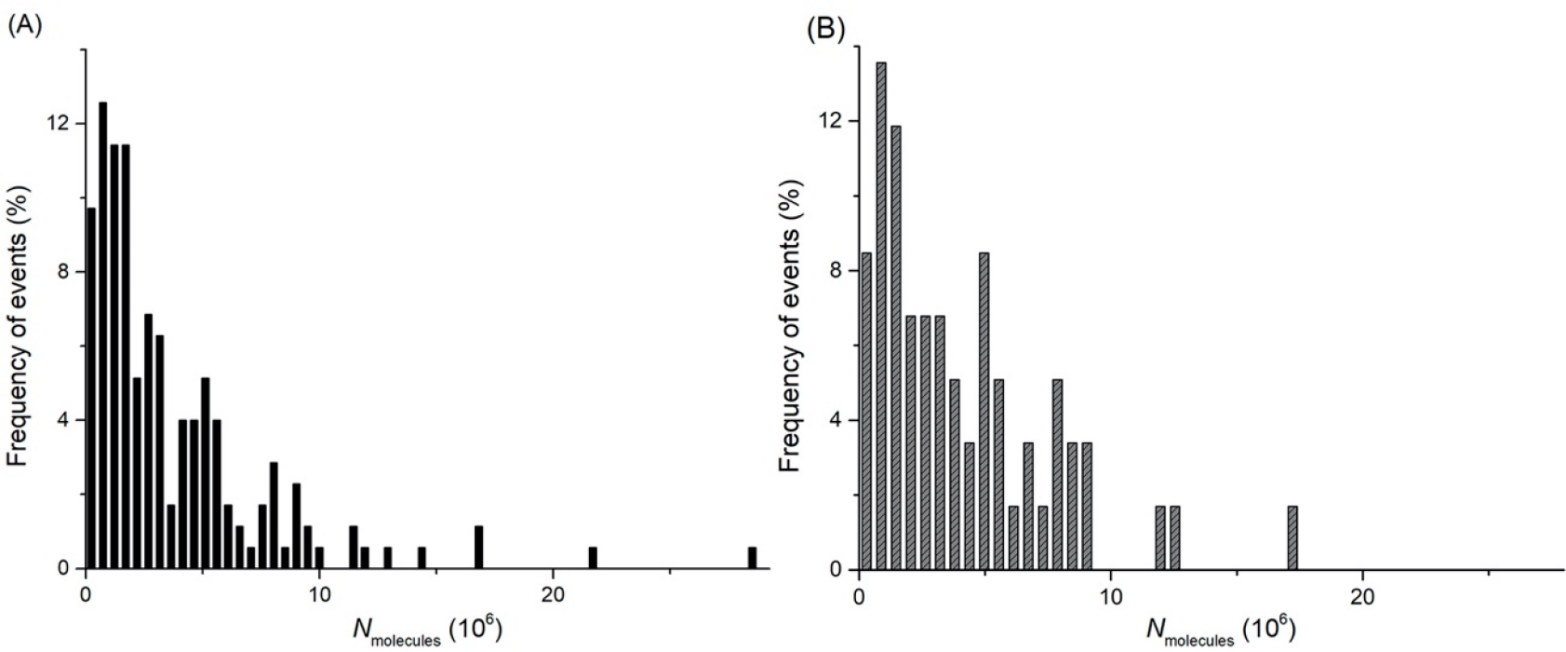
Normalized frequency of events (%) showing (A) the distributions of the molecules from the spikes detected by VIEC (*N*_events_ = 175), and (B) the spikes detected by VIEC correlated with CONF imaging (*N*_events_ = 59) in an isolated vesicle suspension. Bin size: 3.13 × 10^5^ molecules.

For each event, a VIEC spike that occurred within an image frame that corresponded to the disappearance of a vesicle visualized on the CONF image of the same time frame was assigned as a correlated event (discussed below). The normalized histogram of all spikes from the labeled isolated vesicles measured by VIEC alone (Figure 3A) and VIEC correlated with CONF imaging (Figure 3B) showed no significant difference, suggesting the VIEC-correlated CONF strategy can be successfully used to detect the number of molecules released from individual vesicles during the opening process. The average numbers of molecules (*N*_molecules_) for vesicles detected for only VIEC spikes and for VIEC spikes correlated with CONF were 3.68 × 10^6^ and 4.08 × 10^6^, respectively. There is no statistically significant difference (*p* = 0.499) between the *N*_molecules_ from the spikes detected by VIEC (Figure 3A) and spikes detected by VIEC correlated with CONF imaging (Figure 3B), indicating that the CONF-correlated VIEC strategy does not alter our ability to quantify the number of molecules inside single vesicles.

We also observed that not all adsorbed vesicles expel their content. In most cases, the vesicles stayed intact on the electrode surface during the measurement process (10–30 min). As previously shown for liposomes, the rupture and opening processes are faster and more prevalent and suggest all liposomes open upon surface impact.^12^ This contrast leads to an important question of which factors affect the initial pore formation and release of content of vesicles at the electrode. The correlative CONF/VIEC approach used in our study provides a unique opportunity to obtain further insight into this dynamic event.

### Different Opening Patterns of Isolated Vesicles Observed by CONF/VIEC Correlative Imaging

**Figure 4:**
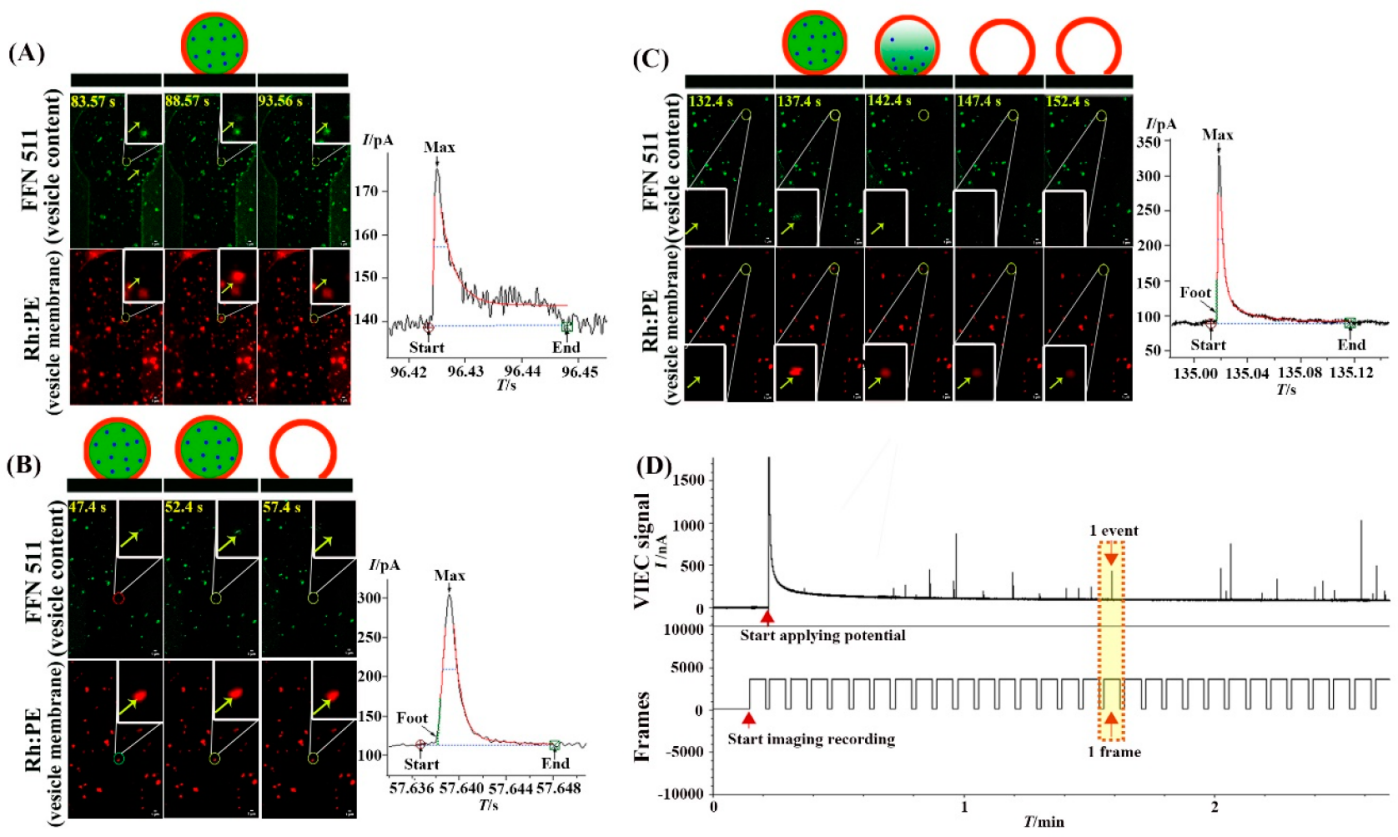
(A–C) Different opening patterns of the isolated vesicles on the electrode surface observed by CONF/VIEC correlation. (D) A typical recording time profile showing the principle of how VIEC spikes and confocal images are correlated.

The combination of VIEC and confocal microscopy provides a unique approach to evaluate how vesicles adsorb and open on the electrode surface. To accomplish this, it is essential to correlate precisely the VIEC events with their corresponding CONF images at the correct time points. An in-house MATLAB code and a triggering tool in the CONF imaging software were used to synchronize the acquisition time in both the VIEC and CONF channels and to track the time frame of individual CONF images for correlation. As shown in Figure 4A, the upper trace shows the VIEC signal vs recording time, while the lower trace shows the CONF signal at the same time frames. FFN511 emits green fluorescence and acts as a monoamine mimic, as it is actively taken up into the vesicles via the vesicle monoamine transporter.^27,28^ This has been used to track vesicle opening events. When a vesicle releases monoamine producing a spike on the VIEC channel, a green fluorescent spot from FFN 511 observed on the CONF channel disappears as the vesicle content is released. Thus, by correlating the CONF images and a VIEC spike in the same time frame, we can use the FFN511 signal to correlate to the VIEC event and determine the location of the vesicle opening at the electrode surface. In addition, the opening process of the vesicle membrane during each release event is simultaneously observed on the CONF channel using the red fluorescence from Rh-PE. The two-color CONF correlative scheme with FFN 511 and Rh:PE allows visualization of the opening process for single vesicles, specifically to observe how a single vesicle approaches, adsorbs on the Au/ITO surface, and expels its content as well as the behavior of the vesicle membrane on the electrode after expelling its content.

Figure 4 shows the three most common opening patterns of vesicles on the electrode surface. These different opening patterns are observed by analysis of vesicle residence time prior to opening. We classified these into (A) vesicles with very short residence time on the electrode surface (≤5 s) followed by the release of content (green fluorescence signal from FFN511 disappearing and a single spike detected by VIEC) and the detachment of the membrane from the electrode (red fluorescence signal from Rh:PE disappearing) (26% of all events); (B) vesicles with short residence time (5–10 s) followed by the release of content and membrane residing on the electrode surface (red fluorescence signal from Rh-PE stayed the same) (30% of all events); (C) vesicles with long residence time (50–520 s) followed by the release of content and membrane residing on the electrode surface (38% of all events). The data including mean residence time and the range of resident times of the vesicles, and the characteristic parameters of VIEC spikes, *T*_half_, *I*_max_, *N*_molecules_, *T*_rise25–75_, and *T*_fall75–25_, from these three patterns are listed in Table 1. Another opening pattern in which the vesicle docked, detached, and returned to the electrode for a short time (15 s, 3 frames) followed by the content release and membrane gradually floating away was observed (Figure S2); however, it only contributed to 6% of 34 events.

**Table 1:** Characteristic Parameters of Different Opening Patterns of Isolated Vesicles^a^

opening patterns	*T*_r_ (s)^b^	*T*_r, range_ (s)^c^	*T*_half_ (ms)^d^	*I*_max_ (pA)^e^	*N*_molecules_ (10^6^)^f^	*T*_rise 25–75_ (ms)^g^	*T*_fall 75–25_ (ms)^h^
**A**	5.00	0–5	2.7	94	1.95	0.4	3.3
**B**	5.62	0–10	7.4	116	3.56	0.9	10.3
**C**	220	50–520	3.6	168	3.54	0.5	4.8

a Data from different vesicle opening patterns: (A) vesicles with short residence times followed by content release and membrane detachment from the electrode surface; (B) vesicles with short residence times followed by content release and membrane remaining on the electrode surface; (C) vesicles with long residence times followed by content release and membrane remaining on the electrode surface. The total numbers of vesicular events measured in groups A, B, and C were 9, 10 and 13, respectively.

b *T*_r_ is the mean residence time from when the vesicle settled on the electrode and released its contents against the electrode.

c *T*_r, range_ is the residence time range used to distinguish each vesicle group.

d *T*_half_ is the mean of width at half-maximum of each peak.

e *I*_max_ is the mean of maximum current for each event.

f *N*_molecules_ is the mean number of molecules oxidized from each vesicle.

g *T*_rise 25–75_ is the rise time for each current transient from 25 to 75% of the peak signal.

h *T*_fall 75–25_ is the mean of the fall time for each current transient from 75 to 25% of the peak signal.

### Vesicles with Larger Catecholamine Content Tend to Adsorb to the Electrode Earlier and Rupture Earlier

**Figure 5:**
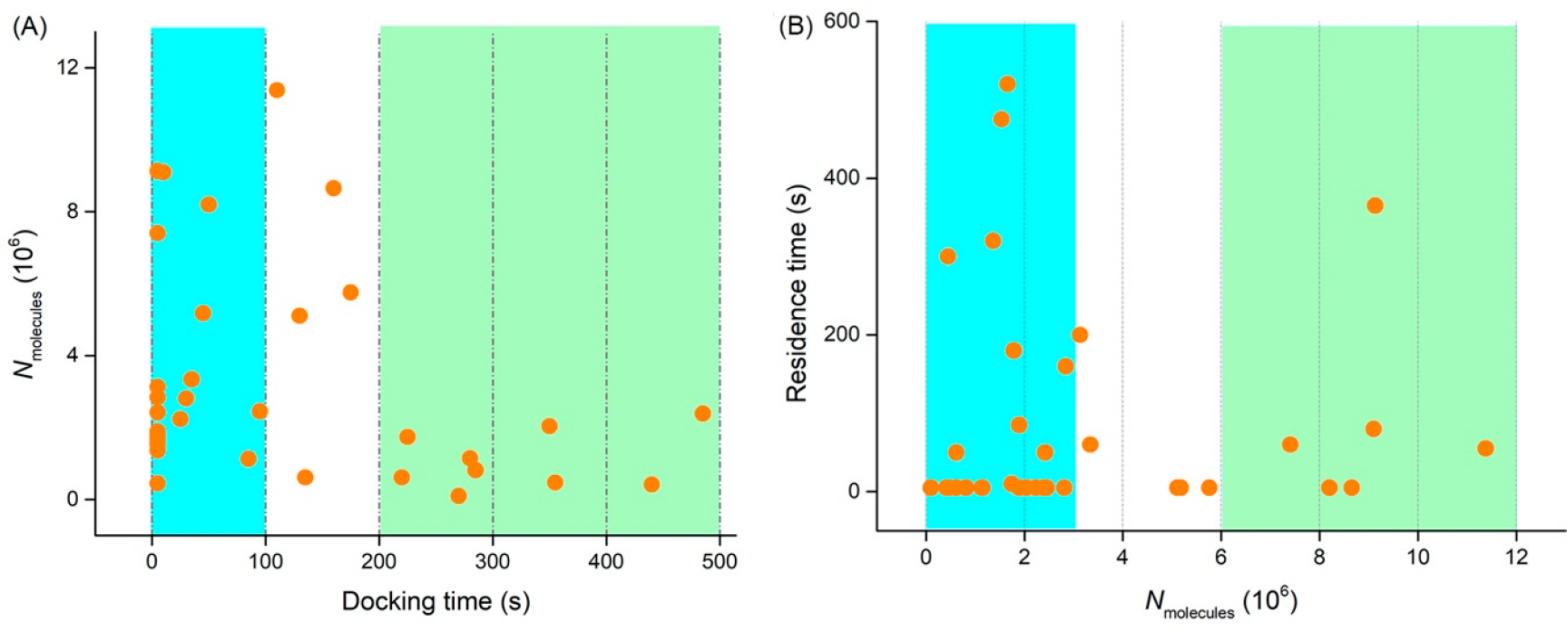
(A) Scatter plots of the number of molecules released from single labeled chromaffin vesicles detected by VIEC vs the corresponding time for a vesicle to dock, “docking time,” on the electrode surface observed by CONF. (B) Scatter plots of the residence time, time before opening, of single chromaffin vesicles observed by CONF vs the number of molecules released from corresponding vesicles detected by VIEC. Number of events: 34.

Important parameters to understand the relation between the vesicle opening mechanism and content released are the time and the frequency that vesicles adsorb to the electrode surface. Figure 5A shows the number of molecules released from labeled vesicles corresponding to their docking time. Five minutes after the vesicle suspension was added, a large number of vesicles were observed to adsorb to the electrode, but a very small number of events were observed with increasing detection time (green region in Figure 5A). From the events occurring during the first 100 s, 65% of events occurred in the first 10 s. By comparing the average *N*_molecules_ of the released content from opening events happening during 0–100 s (blue region) and 200–600 s (green region) in Figure 5A, we note the average *N*_molecules_ from the later green region is significantly decreased (*p* = 0.016) to 2.95 × 10^6^ from 3.50 × 10^6^ for events in the earlier blue region. This suggests that vesicles with a larger amount of catecholamine tend to adsorb to the electrode earlier and open with higher frequency. This might be explained by the vesicles with larger content having a larger physical size and thus larger membrane area, which allows them to more easily adsorb to the electrode surface. Another possibility is that larger size vesicles have more mass and thus settle more quickly onto the electrode surface.

### Vesicle Size Affects Residence Time before Opening and Membrane Residue

The residence time between a vesicle arrival and opening on the electrode surface is correlated with the number of molecules detected from vesicles. In Figure 5B, we notice only vesicles with large content (now shaded in green) tend to have short residence time while only the vesicles with small content (blue region) tend to have long residence time. If we assume the contact area between the vesicle and electrode is proportional to the vesicle content and size, the vesicles with large content (size) will have a larger contact area with the electrode. Electron micrographs of vesicles trapped on an electrode surface suggest vesicles flatten when adsorbed.^8^ The potential used for electrooxidation, when using the membrane thickness of 5 nm, provides a field in the range used for membrane electroporation.^12^ This will lead to a higher probability for the initial pore formation driven by electroporation for VIEC measurement and thus a shorter residence time before opening, consistent with a hypothesis in a previous study.^35^ The hypothesis is that membrane proteins randomly move until a portion of membrane is free to wobble near to the electrode, exposing it to a field large enough for electroporation. The larger the contact area, the better the chance to expose a section of membrane and initiate electroporation. Although this explains why larger vesicles tend to have shorter residence time and only small vesicles tend to have long residence time, it is not clear why some smaller vesicles have short residence times. It is possible that pore formation by electroporation depends on both vesicle size and the protein distribution on the membrane near the electrode surface. The membrane proteins might form a barrier restricting the lipid membrane from reaching the electrode surface, and these need to move away before the electroporation occurs.^12^ The smaller the vesicles are, the larger this barrier becomes for the occurrence of electroporation, as the distance between the vesicle membrane and the electrode surface caused by the proteins should increase relative to the vesicle size and membrane curvature.

It is also interesting that a residue of the vesicle membrane for large vesicles is left on the electrode after the vesicle releases its content. Although it is possible that some membrane sections left behind are not observed owing to photobleaching, it seems unusual that this would have a vesicle size dependence. Still, it cannot be ruled out. If we assume we are not photobleaching, and this is not usually an issue in CONF, and as the vesicular catecholamine content is generally correlated with vesicle size,^37,38^ we can speculate that larger size vesicles attach more strongly to the electrode surface, perhaps due to their increased contact area with the electrode. Thus, vesicles with large content and thus larger size provide stronger intermolecular forces compared to small vesicles, making the membranes of large vesicle more difficult to dissolve back into the solution.

## Conclusions

In summary, we present a novel hybrid method, correlative CONF/VIEC, for visualization and quantification of the dynamic opening process of single isolated vesicles during their docking and opening at an electrode surface. This is carried out with an in-house 5-nm-thick, gold-modified ITO microelectrode, and the method provides complementary optical and electrochemical information for understanding the nature of the opening process and how it relates to the content and the size of vesicles. Three main opening patterns have been observed regarding to the residence time and retention time of the vesicles before and after content release, respectively. The data collected from both small vesicles and large vesicles shows that the larger vesicles with high catecholamine content tend to arrive and dock at the electrode faster followed by a shorter residence time before opening and releasing their content, and their membranes remain on the electrode surface longer. This demonstrates that the vesicle adsorption time, residence time before releasing, and the vesicle membrane behavior after release are dependent on the vesicle content and/or size.

The main point of this work has been to identify how vesicles absorb and open on an electrode surface to further understand VIEC. We cannot be certain that this will generalize to processes occurring during exocytosis in living cells, but we can speculate that the trends with vesicle opening will be similar in the sense that the free energy of the vesicle membrane is the same in both cases, although this is likely to be skewed by the differences in adsorption to the gold surface. We feel this is not likely to dominate as the processes seem to be similar from the electrochemistry to those occurring at carbon electrodes. So, at least in part, these processes might be generalizable to a vesicle in a presynaptic terminal. These fundamental findings should be useful for further investigation of vesicle related biological processes, particularly vesicle transport, fusion-pore dynamics, and exocytosis.

## Supporting Information Available

Figure S1. Scanning probe microscopy characterization for a bare ITO-coated coverslip and a 5 nm gold thin film deposited onto ITO glass. Figure S2. Isolated vesicle opening pattern observed by confocal microscopy and corresponding VIEC spike (PDF)
