# Supplementary material for: Dynamic Visualization and Quantification of Single Vesicle Opening and Content by Coupling Vesicle Impact Electrochemical Cytometry with Confocal Microscopy
Source: ACS Meas Sci Au. 2021 Aug 9;1(3):131–8. doi: 10.1021/acsmeasuresciau.1c00021 (PMC8679085; doi:10.1021/acsmeasuresciau.1c00021)
Supplement: Supplementary file 1 — tg1c00021_si_001.pdf [file tg1c00021_si_001.pdf]

Dynamic visualization and quantification of single vesicle opening and content by coupling vesicle impact electrochemical cytometry with confocal microscopy

Ying-Ning Zheng, Tho D. K. Nguyen, Johan Dunevall, Nhu T. N. Phan*, Andrew G. Ewing*

Department of Chemistry and Molecular Biology, University of Gothenburg, Kemivägen 10, 41296 Gothenburg, Sweden

Supporting information

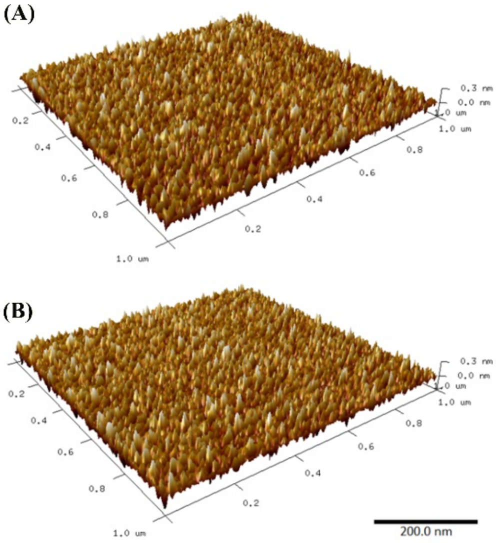

Figure S1. Scanning probe microscopy characterization (A) for a bare ITO coated cover slip and (B) a 5 nm gold thin film deposited onto ITO glass.

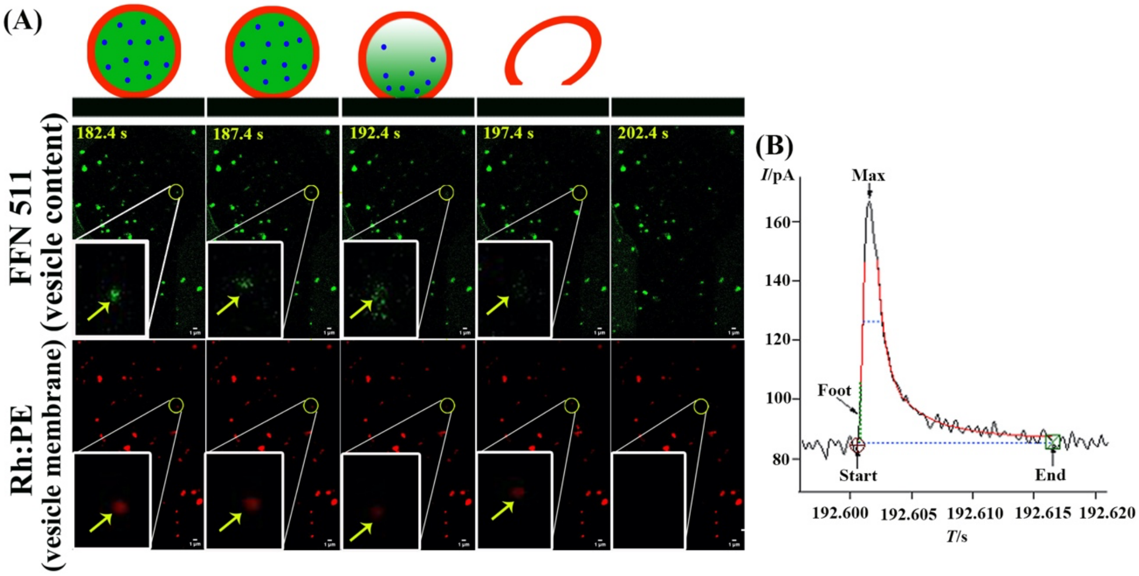

Figure S2. (A) Isolated vesicle opening pattern observed by confocal microscopy and (B) corresponding VIEC spike.
